# Myasthenia gravis - a retrospective analysis of e-mail inquiries made to a patient organisation and specialized center to uncover unmet needs from patients and caregivers

**DOI:** 10.1186/s12883-022-02981-y

**Published:** 2022-12-07

**Authors:** Maike Stein, Sarah Hoffmann, Lea Gerischer, Frauke Stascheit, David Legg, Andreas Meisel, Sophie Lehnerer

**Affiliations:** 1 Charité – Universitätsmedizin Berlin, corporate member of Freie Universität Berlin and Humboldt-Universität zu Berlin, Department of Neurology with Experimental Neurology, Charitéplatz 1, 10117 Berlin, Germany; 2 Charité – Universitätsmedizin Berlin, corporate member of Freie Universität Berlin and Humboldt-Universität zu Berlin, Department of Neurology with Experimental Neurology, NeuroCure Clinical Research Center, Charitéplatz 1, 10117 Berlin, Germany; 3 Charité – Universitätsmedizin Berlin, Center for Stroke Research Berlin, Charitéplatz 1, 10117 Berlin, Germany; 4 Berlin Institute of Health at Charité – Universitätsmedizin Berlin, Digital Health Center, Charitéplatz 1, 10117 Berlin, Germany

**Keywords:** Myasthenia gravis, Counselling, Patient organisation, Unmet needs, E-mail, Social legislation, COVID-19, Caregiver, Medical inquiries, Specialized care

## Abstract

**Background and aims:**

Myasthenia Gravis requires expert treatment from specialized neurologists. In Germany, this treatment is mainly provided by 18 Integrated Myasthenia Centers (iMZ) accredited by the German Myasthenia Gravis Association (DMG). The DMG is a large and well-organized patient organisation that is regarded as a trusted source for disease-specific information. The aim of this study was to analyse the type of requests that each of these institutions receives in order to identify any potential unmet needs regarding the availability of advice for patients and caregivers. This data can then be used in further research to tailor modern digital communication tools to the specific needs of MG patients.

**Methods:**

Counselling requests sent via e-mail to both institutions were extracted for defined examination periods and divided into a period ‘before COVID-19 pandemic’ (01.07.2019–31.12.2019) and ‘during COVID-19 pandemic’ (01.07.2020–31.12.2020). Requests were then analysed using four main categories: medical requests, organisational issues, COVID-19 and social legislation inquiries.

**Results:**

One thousand seven hundred eleven requests for advice were addressed to DMG and iMZ Charité. Most inquiries directed to the DMG (47%; *n = 750*) were related to medical issues, most frequently to side effects of medications (*n = 325*; 20%) and questions about treatment (*n = 263*; 16%), followed by inquiries regarding organisational issues (26%; *n = 412*). About half of the inquiries (*n = 69*; 58%) to the iMZ Charité were related to medical issues and almost one in three inquiries concerned organisational issues (*n = 37*; 30%). About one in ten inquiries concerned socio-legal matters (iMZ: *n = 7*; 6% and DMG: *n = 177*; 11%). During the pandemic, COVID-19 related issues accounted for 8% (*n = 6*) of inquiries at iMZ, and 16% (*n = 253*) at DMG.

**Conclusions:**

MG sufferers have a high demand for timely advice. In the current setting, they address their requests to both iMZs and the DMG via e-mail. Our findings confirm that the DMG is highly trusted by patients and caregivers and is used to obtain second opinions. A relevant proportion of requests to the iMZ could be answered more effectively through standardized responses or improved process management. The implementation of modern digital solutions, including telemedicine, for communication between patient and specialist should be evaluated in further research.

**Supplementary Information:**

The online version contains supplementary material available at 10.1186/s12883-022-02981-y.

## Background

Myasthenia gravis (MG) is a rare autoimmune disease (prevalence 15–20/100.000) in which specific antibodies target the neuromuscular junction, leading to fatigability of the ocular, bulbar and skeletal muscles [1–3]. Due to the rarity of the condition and complexity of symptoms, diagnosis and treatment of the disease is often limited to specialized neurologists. In Germany, this care is primarily provided by 18 specialized integrated Myasthenia Gravis Centers (iMZ), certified by the German Myasthenia Association (DMG) [4]. Together, these centers form an expert information network that can facilitate and deliver a more integrated and responsive form of health care. Several projects initiated by this network and the DMG focus on the patient’s perspective and identification of potential unmet needs. The aim of these projects is to create a basis for a more patient-centric treatment and to help health professionals caring for MG patients in joint decision-making.

The specialized neurologists of the iMZs offer information, advice, and individualized counselling for MG patients on a range of subjects including: healthcare services, medication including compliance and interactions, health promoting lifestyle and social law inquiries. Complementing the medical care, the DMG provides a comprehensive information and networking service available to all patients, caregivers, and health care providers. The patient organisation has around 3400 members and its responsibilities include advocacy for MG patients and their families, as well as public relations and support in social matters.

Counselling requests reflect unmet needs of patients and their caregivers, but have not been well studied among patients with MG. The aim of this study was to analyse the type of requests received by the two institutions (DMG and iMZ) to improve the understanding of health-seeking behaviours and to identify potential unmet needs related to the availability of counselling for patients and caregivers. While a difference in the requests made to both institutions is expected given their respective focus, COVID-19 pandemic inevitably shifted some of the previous services in the health sector to the digital sector. This data can then be used in further research to tailor modern digital communication tools to the specific needs of MG patients to provide a more integrated form of care.

## Methods

To identify what was important to MG service users (patients, caregivers, healthcare professionals), the content of e-mails sent to the DMG and iMZ Charité – Universitätsmedizin Berlin was extracted and categorised. E-mails sent to the iMZ and the DMG between dates 01.07.2019 and 31.12.2019 (‘before COVID-19 pandemic’) and 01.07.2020 and 31.12.2020 (‘during COVID-19 pandemic’) were analysed. Importantly, one e-mail could feature more than one request. Inquiries to the DMG were sent to e-mail address info@dmg-online.de and were either answered by their office or the medical advisory board, depending on the type of question asked. E-mails to the iMZ Charité – Universitätsmedizin Berlin were received via a functional e-mail address (myasthenie-notfallberatung@charite.de), started in March 2020 (just after the start of the pandemic), and two physicians’ private work e-mail addresses at the iMZ Charité – Universitätsmedizin Berlin.

Rather than coding directly from the e-mails, the principal investigator designed a standardised data extraction form (see Supplementary material) in order to record key data points including the contacted institution, author characteristics and the type and frequency of request made. Requests were then categorised into one of four main categories designed by the primary researcher. These included medical inquiries, organisational issues, social legislation inquiries and COVID-19 related questions. Each category had a distinct set of subcategories which detailed more specific question classification. To ensure reliability to the categorisation system, the raw data was extracted and coded by two independent raters. After rater 1 extracted all the relevant data, rater 2 also extracted and categorised all data of iMZ Charité e-mails. Any conflict was then resolved by consensus. We have not done so for the patient organisation due to limited time resources. The categorisation system for the topics of interest for the iMZ Charité can be deemed as reliable as two independent researchers produced comparable results as they applied the same categorisation system for the iMZ requests.

### Statistical analysis

All data were analysed in Excel (version 2002) from Microsoft Office 365 ProPlus (version 2203). Frequencies were calculated and data was presented as counts and percentages. Z tests were used to establish the impact of COVID-19 on the DMG and iMZ question categories and *P* values were calculated to determine significance of any percentage changes (*P* = < 0.05).

## Results

During the defined examination periods a total of 1711 consultation requests were made to both institutional bodies the iMZ and DMG. A total of 1592 written consultation requests were sent to the DMG between 01.07.2019 and 31.12.2019 (‘before COVID-19 pandemic’, *n = 633*) and 01.07.2020 and 31.12.2020 (‘during COVID-19 pandemic’, *n = 959*) (Table 1). A total of 110 consultations requests were sent to the iMZ between 01.07.2019 and 31.12.2019 (‘before COVID-19 pandemic’, *n = 37*) and between 01.07.2020 and 31.12.2020 (‘during COVID-19 pandemic’, *n* = 73). In most of the requests to the iMZ, only one question was asked. In a few cases, one e-mail request contained multiple questions, resulting in a higher total of 119 consultation requests than e-mails (Table 2).

**Table 1:**
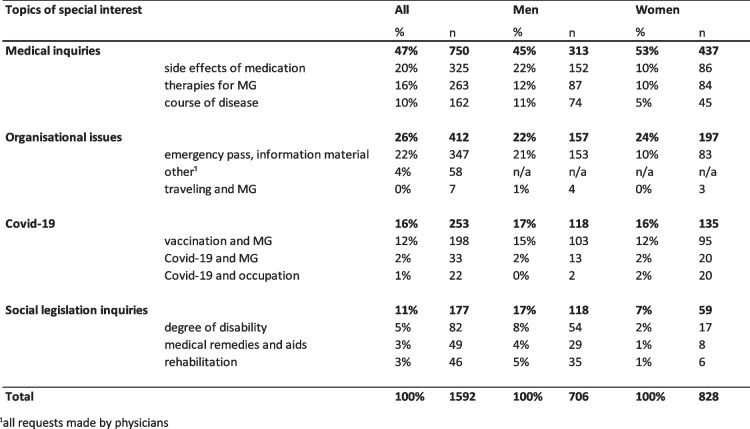
Overview and topics of interest and inquiries to the DMG

**Table 2:**
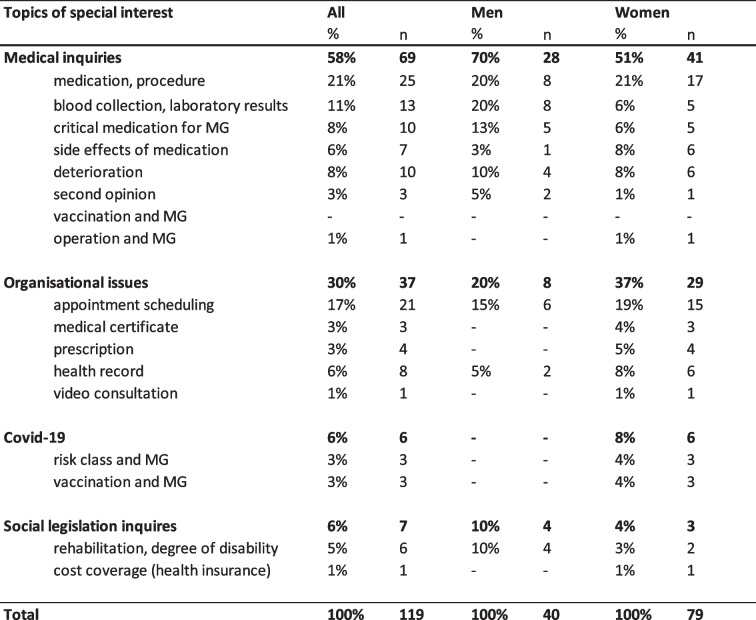
Overview of topics of interest of inquiries to the iMZ

Inquiries to the DMG were mainly made by patients or caregivers (‘before COVID-19 pandemic’: *n = 608*; 95%; ‘during COVID-19 pandemic’: *n = 926*; 97%). In 58 cases (4%) inquiries were made by healthcare professionals. Of all inquiries to the DMG, 706 (44%) were male and 828 (52%) were female. In 58 cases (4%) no gender was documented (all of them were from healthcare professionals).

Of the individuals contacting the iMZ 66 (60%) were female. Most individuals contacting the iMZ were MG patients or caregivers (*n = 106*; 96%) and significantly fewer health care professionals (*n = 4*; 4%).

### Topics of interest and inquiries to the DMG

The majority of inquiries from medical service users to the DMG were related to questions about medical topics (*n = 750*; 47%). Every fourth inquiry was about organisational issues (*n = 412*; 26%), followed by 253 inquiries related to COVID-19 (16%). About every tenth inquiry (*n = 177*; 11%) was about social law matters (Table 1). The most common subcategories for medical questions were about drug interactions and side effects of medication (*n = 325*; 20%) and general information about therapies for MG (*n = 263*; 16%). 162 individuals (10%) needed information about the course of the disease.

Organisational inquiries mainly included the request for an emergency pass (*n = 347*; 22%). All inquiries from healthcare professionals (*n = 58*; 4%) asked for information material.

Many of the counselling requests regarding COVID-19 were about the need for information about SARS-CoV-2 vaccination (*n = 198*; 12%). In 22 cases (1%) patients or care givers had questions regarding occupational activity and COVID-19. 33 individuals (2%) needed other information on COVID-19 and MG (e.g., individual risk group for a severe disease progression in case of SARS-CoV-2 infection).

Social legislations inquiries included topics about the degree of disability (*n = 82*; 5%), medical remedies and aids (*n = 49*; 3%) and rehabilitation (*n = 46*; 3%). No questions about lifestyle (e.g., diet), networking/local events or recommendations for specialists were asked.

If one compares the requests to the DMG in the periods before and during COVID-19, the percentage difference between the ‘before COVID-19’ and ‘during COVID-19’ periods are not statistically significant. Therefore, COVID-19 did not change how users interacted with the DMG, outside of COVID-19 becoming a new topic (Table 3). The detailed data can be found in the supplementary material.

**Table 3:**
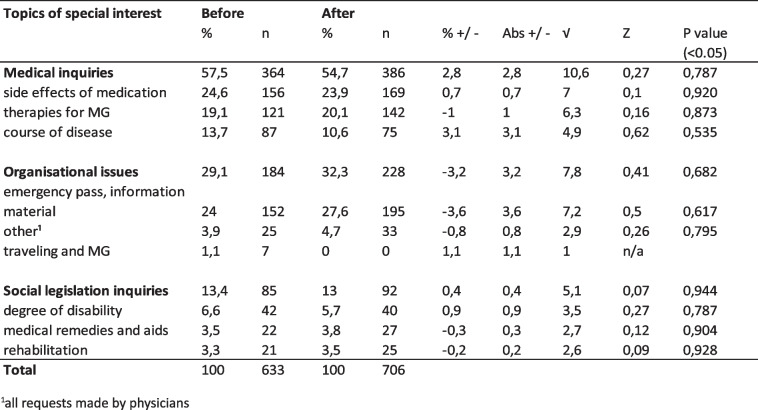
Significance tests of inquiries to DMG before and during the COVID-19 pandemic

### Topics of interest and inquiries to the iMZ

About half of all inquiries to the iMZ related to questions about medical topics (*n =* 69; 58%), followed by questions about organisational issues (*n =* 37; 30%) and social legislation inquiries (*n =* 7; 6%). In the time period ‘during Covid-19 pandemic’ in six cases (8%) patients had questions related to COVID-19.

The medical inquiries were divided into questions about medication and procedure (*n =* 25; 21%), blood collection and laboratory results (*n* = 13; 11%), critical medication for MG (*n =* 10; 8%), side effects of medication (*n =* 7; 6%) and questions regarding clinical deterioration (*n =* 10; 8%). In three cases (3%) patients requested an independent second opinion and one (1%) asked for information on operations (thymectomy) regarding MG. Concerning organisational inquiries, the most common question was about appointment scheduling (*n =* 21; 17%). Other frequent requests were requirements for a health record (*n =* 8; 6%), medical certificate (*n = 3*; 3%) and prescription (*n = 4*; 3%). Social legislation inquiries included questions about rehabilitation and for a medical report or information regarding type or degree of the disability (*n = 7*; 6%).

If one compares the requests to the iMZ in the periods before and during COVID-19, the percentage difference between the ‘before COVID-19’ and ‘during COVID-19’ periods are not statistically significant. Therefore, COVID-19 did not change how users interacted with the iMZ (analogous to the significance test of the DMG), outside of COVID-19 becoming a new topic (Table 4). The detailed data can be found in the supplementary material.

**Table 4:**
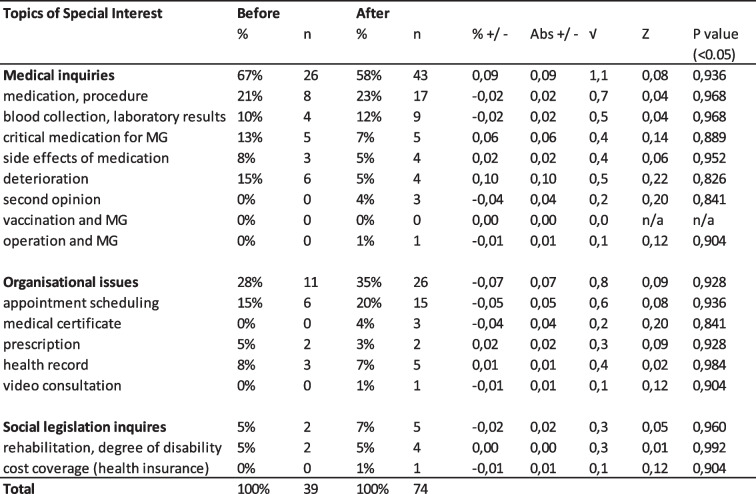
Significance tests of inquiries to iMZ before and during the COVID-19 pandemic

## Discussion

In this retrospective study counselling requests directed to a specialized myasthenia gravis center and to a patient organisation were analysed to identify potential unmet needs of patients, caregivers or healthcare professionals.

The main findings of the study were that the majority of inquiries (~ 50%) to the iMZ and DMG were medical questions and were mainly made from patients and caregivers. About a third of the requests were organisational requests that could potentially be managed in a better way. Also, COVID-19 did not change the nature of inquiries to the iMZ and DMG, except that COVID-19 became a new topic.

There is clearly a high need for advice, both in the iMZ as well as the patient organisation with specialized services. Requests to the DMG served at least in part to obtain a second opinion. It is noteworthy that not only social law inquiries or requests for information material were directed to the DMG (e.g., on COVID-19, emergency pass, vaccinations) but in nearly half of all inquiries medical questions were asked (e.g., course of disease or side effects of medication). This demonstrates a high level of trust in the quality of advice provided by the DMG. However, it remains an open question whether these requests were made by patients who are not treated at an iMZ. In this case, it could potentially indicate insufficient information for patients and caregivers from the outpatient area and/or raise the question of satisfaction with the treatment. In a large cross-sectional study including 1660 MG patients (all being DMG members), we found that despite drug therapy 20% could not perceive any improvement in myasthenic symptoms [5]. Therefore, non-response to treatment could also be a reason for dissatisfaction and generate the need for further information. Other possible reasons could be the perceived lack of access to experienced neurologists. It is likely that the majority of persons addressing the DMG are also members of the DMG and it is conceivable that more severely than mildly affected MG patients might register as members of a patient organisation [6–8]. Furthermore, more inquiries to both institutions were made by women indicating a higher need for advice. Even though there is a higher prevalence among women for MG, several studies have demonstrated that women have a higher overall burden of disease [6, 9, 10]. In addition, the disease itself has a strong impact on family planning and pregnancy [11]. However, this was not reflected in the topics of (medical) requests. It is possible that sensitive issues like pregnancy and sexuality are rather addressed in person during appointments than via e-mail. Surprisingly, there were hardly any requests about lifestyle or travel.

When compared to the patient organisation more than half (58%) of the inquiries to the iMZ were medical questions, half of which required a direct exchange with the specialist for clinical procedure or clinical deterioration. Patients at the iMZ could be supposed to have a more complicated course of the disease and have established access to experts via in-patient visits already. Of the almost one third of organisational inquiries to the iMZ, more than half were questions about appointments or medical records. This indicated that there is potential for improvement in the organisational processes since this is the core task of the administration of the outpatient clinics and not primarily a medical task.

During the pandemic, patients and caregivers regularly contacted the DMG with individual advice requests, mainly on the topics such as SARS-CoV-2 vaccination or occupation and COVID-19. The medical board of the DMG therefore published a recommendation and a constantly updated guideline related to COVID-19, vaccination and MG on the DMG website, which should serve as a decision-making aid for patients and healthcare practitioners [12]. Since MG patients are considered risk patients for a more severe disease course in case of SARS-CoV-2 infection [13], questions directed to the iMZ mainly revolved around their individual risk group and/or a medical certificate for prioritized vaccination.

As for social law matters, the results indicate that both bodies play a role in providing advice for social legislation inquiries (DMG: 11%; iMZ 6%). Inquiries directed to the DMG included requests for medical remedies and aids (e.g., wheelchair). This was not relevant in the inquiries of the iMZ, but these concerns could potentially also be discussed and issued during the personal consultation hours. Almost all social legislation inquiries to the iMZ were about rehabilitation or supporting medical reports for applying or worsening for the degree of disability pass. In the application process, these medical statements can be decisive for pension claims. Doctors are called upon by the pension office (Versorgungsamt) to comment and can support patients in the process. Inquiries relating to social law matters were made to both institutions more frequently by men in particular (DMG: 17% vs 7%; iMZ 10% vs 4%). From a clinical perspective it is well known that the majority of MG patients experiences limitations regarding employment due to MG such as incapacity of work or recurrent occupational disability. While only a few studies have examined the ability of MG patients to work [14–16], there is evidence to suggest that MG patients are confronted with far-reaching social legislation effects which is shown not only in our results but also in a large German study in cooperation with DMG [5, 17]. The study found that only 25.5% of the MG patients worked more than 15 h per week and almost 70% had no labour market participation. The main reasons were age and disability related retirement [17]. This detrimental effect on labour market participation has also been confirmed by an Australian study which showed that 40% stopped work due to MG and nearly 20% had to change occupation [15]. This impact is also likely to be worsened by a low level of social support. Lehnerer et al. (2021) found that low social support was reported by one fifth of the study participants and that these affected patients also showed more difficulties in daily life activities, more symptoms of anxiety and depression and experienced a lower quality of life [5]. In combination with the disease itself, this is likely to exacerbate patients’ difficulties with functional abilities. This highlights again the importance of institutional advice and support in offering a holistic range of counselling for patients and caregivers with specialized services.

It should be noted that the study has some methodological limitations. First of all, the data was not correlated with further clinical parameters such as disease severity (MGFA) and no information was conducted about unique and repeat service users. It is also likely that young to middle-aged patients in particular contacted us via e-mail. The difference in the number of inquiries to both institutions could be explained by the fact that the functional e-mail address of the iMZ only started in March 2020, shortly after the beginning of the pandemic and the number of inquiries has gradually increased. In addition, a larger team of neurologists work at the iMZ Berlin, but only the inquiries to two of the physicians’ personal work e-mail address was included into the analysis, therefore a cumulatively higher number of inquiries occurred during the investigation period. Because the author only started working in the myasthenia gravis outpatient clinic at the end of 2019, requests to another colleague from the iMZ and co-author were analysed for the period ‘before COVID-19 pandemic’.

## Conclusion

In summary, a clear need for advice of patients, caregivers and healthcare professionals that is not reflected in the routine examinations can be identified. Since the main feature of MG is a fluctuating, potentially life-threatening muscle weakness, continuous outpatient medical therapy adjustments and concepts of clinical surveillance are required. This is also reflected in the high number of medical inquiries to both organisations. In general, a high number of requests (~ 50%) could have been answered with standardized responses or improved steering of processes. However, about 25% of medical requests still needed direct communication with a specialist. Directing these kinds of requests via e-mail to a patient organisation (or even the specialists) might lead to dangerous delays in obtaining the necessary information. Therefore, modern digital communication tools tailored to the specific needs of MG patients are warranted [18, 19]. Further research is needed to evaluate the implementation of these tools, including telemedicine, for communication between patient and specialist.

## Supplementary Information

**Additional file 1.**

## Data Availability

All data generated or analysed during this study are included in this published article.

## Abbreviations

MG: Myasthenia gravis
iMZ: Myasthenia Gravis Centers
DMG: German Myasthenia Association

## References

[1] Huijbers MG, Marx A, Plomp JJ, Le Panse R, Phillips WD (2022). Advances in the understanding of disease mechanisms of autoimmune neuromuscular junction disorders. Lancet Neurol.

[2] Verschuuren JJ, Palace J, Murai H, Tannemaat MR, Kaminski HJ, Bril V (2022). Advances and ongoing research in the treatment of autoimmune neuromuscular junction disorders. Lancet Neurol.

[3] Gilhus NE, Tzartos S, Evoli A, Palace J, Burns TM, Verschuuren J (2019). Myasthenia gravis. Nat Rev Dis Primers.

[4] Bungard S, Rohn H, Dobler K (2011). Certification of myasthenia centres: developing and implementing a certification procedure for patient organisations. Z Evid Fortbild Qual Gesundhwes.

[5] Lehnerer S, Jacobi J, Schilling R, Grittner U, Marbin D, Gerischer L, et al. Burden of disease in myasthenia gravis: taking the patient's perspective. J Neurol, 269(6):3050–63. 10.1007/s00415-021-10891-1 PMC9120127 34800167

[6] Boldingh MI, Dekker L, Maniaol AH, Brunborg C, Lipka AF, Niks EH (2015). An up-date on health-related quality of life in myasthenia gravis -results from population based cohorts. Health Qual Life Outcomes.

[7] Jeong A, Min JH, Kang YK, Kim J, Choi M, Seok JM (2018). Factors associated with quality of life of people with myasthenia gravis. PLoS One.

[8] Boscoe AN, Xin H, L'Italien GJ, Harris LA, Cutter GR (2019). Impact of refractory myasthenia gravis on health-related quality of life. J Clin Neuromuscul Dis.

[9] Padua L, Evoli A, Aprile I, Caliandro P, Mazza S, Padua R (2001). Health-related quality of life in patients with myasthenia gravis and the relationship between patient-oriented assessment and conventional measurements. Neurol Sci.

[10] Dong D, Chong MK, Wu Y, Kaminski H, Cutter G, Xu X (2020). Gender differences in quality of life among patients with myasthenia gravis in China. Health Qual Life Outcomes.

[11] Ohlraun S, Hoffmann S, Klehmet J, Kohler S, Grittner U, Schneider A (2015). Impact of myasthenia gravis on family planning: how do women with myasthenia gravis decide and why?. Muscle Nerve.

[12] Blaes F. M A, Meisel A., Schroeter M., Kaiser J., Schara-Schmidt U., Hagenacker T., Totzeck A., Della Marina A., Wiendl H., Keller C., Ruck T., Krämer-Best, H., Dersch R., Knop KC., Urban P., Thayssen G., Thieme A., Kalischewski P., De-Hyung L., Jacobi C., Zschüntzsch J. Aktuelle Informationen zur COVID-19-Impfung von Patienten mit myasthenen Syndromen 2021 [updated 30.09.2021. Available from: https://dmg-online.de/pdf/4._Aktualisierung_COVID-19_Impfung_bei_MG_.pdf.

[13] Abbas AS, Hardy N, Ghozy S, Dibas M, Paranjape G, Evanson KW (2022). Characteristics, treatment, and outcomes of myasthenia gravis in COVID-19 patients: a systematic review. Clin Neurol Neurosurg.

[14] Frost A, Svendsen ML, Rahbek J, Stapelfeldt CM, Nielsen CV, Lund T (2016). Labour market participation and sick leave among patients diagnosed with myasthenia gravis in Denmark 1997-2011: a Danish nationwide cohort study. BMC Neurol.

[15] Blum S, Lee D, Gillis D, McEniery DF, Reddel S, McCombe P (2015). Clinical features and impact of myasthenia gravis disease in Australian patients. J Clin Neurosci.

[16] Harris L, Aban IB, Xin H, Cutter G (2019). Employment in refractory myasthenia gravis: a Myasthenia Gravis Foundation of America registry analysis. Muscle Nerve.

[17] Twork S, Wiesmeth S, Klewer J, Pohlau D, Kugler J (2010). Quality of life and life circumstances in German myasthenia gravis patients. Health Qual Life Outcomes.

[18] Ricciardi D, Casagrande S, Iodice F, Orlando B, Trojsi F, Cirillo G (2021). Myasthenia gravis and telemedicine: a lesson from COVID-19 pandemic. Neurol Sci.

[19] Guidon AC, Muppidi S, Nowak RJ, Guptill JT, Hehir MK, Ruzhansky K (2021). Telemedicine visits in myasthenia gravis: expert guidance and the myasthenia gravis Core exam (MG-CE). Muscle Nerve.

