# Supplementary material for: Myasthenia gravis - a retrospective analysis of e-mail inquiries made to a patient organisation and specialized center to uncover unmet needs from patients and caregivers
Source: BMC Neurol. 2022 Dec 7;22:455. doi: 10.1186/s12883-022-02981-y (PMC9727989; doi:10.1186/s12883-022-02981-y)
Supplement: Supplementary file 1 — Additional file 1. [file 12883_2022_2981_MOESM1_ESM.docx]

**Supplementary material**

1. Data extraction form iMZ (Time period ‘before COVID-19 pandemic‘: 01.07.2019-31.12.2019)

| **Topics of special interest** | **All (%; *n*)** | **Men (%; *n*)** | **Women (%; *n*)** |
| --- | --- | --- | --- |
| **Medical requests** |  |  |  |
| Medication, procedure |  |  |  |
| Blood collection, laboratory results |  |  |  |
| Side effects of medication |  |  |  |
| Second opinion |  |  |  |
| Operation and MG |  |  |  |
| Critical medication for MG |  |  |  |
| Deterioration |  |  |  |
| Vaccination and MG |  |  |  |
|  |  |  |  |
| **Organisational issues** |  |  |  |
| Appointment scheduling |  |  |  |
| Medical certificate |  |  |  |
| Prescription |  |  |  |
| Health record |  |  |  |
| Other |  |  |  |
| Video consultation |  |  |  |
|  |  |  |  |
| **Social legislation inquiries** |  |  |  |
| Rehabilitation, degree of disability |  |  |  |
| Remedies and aids |  |  |  |
| Cost coverage |  |  |  |

1. Data extraction form iMZ (Time period ‘during Covid-19 pandemic’:01.07.2020-31.12.2020)

| **Topics of special interest** | **All (%; *n*)** | **Men (%; *n*)** | **Women (%; *n*)** |
| --- | --- | --- | --- |
| **Medical requests** |  |  |  |
| Medication, procedure | - |  |  |
| Blood collection, laboratory results |  |  |  |
| Side effects of medication |  |  |  |
| Second opinion |  |  |  |
| Operation and MG |  |  |  |
| Critical medication for MG |  |  |  |
| Deterioration |  |  |  |
| Vaccination and MG |  |  |  |
|  |  |  |  |
| **Organisational issues** |  |  |  |
| Appointment scheduling |  |  |  |
| Medical certificate |  |  |  |
| Prescription |  |  |  |
| Health record |  |  |  |
| Other |  |  |  |
| Video consultation |  |  |  |
|  |  |  |  |
| **COVID-19 related requests** |  |  |  |
| Risk class and MG |  |  |  |
| SARS-CoV-2 vaccination and MG |  |  |  |
|  |  |  |  |
| **Social legislation inquiries** |  |  |  |
| Rehabilitation, degree of disability |  |  |  |
| Remedies and aids |  |  |  |
| Cost coverage |  |  |  |

3. Data extraction form DMG (Time period ‚before COVID-19 pandemic‘ 01.07.2019-31.12.2019)

| **Topics of special interest** | **All (%; *n*)** | **Men (%; *n*)** | **Women (%; *n*)** |
| --- | --- | --- | --- |
| **Medical inquiries** |  |  |  |
| Course of disease |  |  |  |
| Therapies for MG |  |  |  |
| Side effects of medication |  |  |  |
|  |  |  |  |
| **Organisational issues** |  |  |  |
| Emergency pass, information material |  |  |  |
| Membership DMG |  |  |  |
| Events/networking |  |  |  |
| Lifestyle and MG |  |  |  |
| Traveling and MG |  |  |  |
|  |  |  |  |
| **Social legislation inquiries** |  |  |  |
| Rehabilitation, degree of disability |  |  |  |
| Remedies and Aids |  |  |  |

4. Data extraction form DMG (Time period ‚during COVID-19 pandemic‘ 01.07.2020-31.12.2020)

| **Topics of special interest** | **All (%; *n*)** | **Men (%; *n*)** | **Women (%; *n*)** |
| --- | --- | --- | --- |
| **Medical inquiries** |  |  |  |
| Course of disease |  |  |  |
| Therapies for MG |  |  |  |
| Side effects of medication |  |  |  |
|  |  |  |  |
| **COVID-19 related requests** |  |  |  |
| COVID-19 and MG |  |  |  |
| SARS-CoV2 vaccination and MG |  |  |  |
| COVID-19 and occupation |  |  |  |
|  |  |  |  |
| **Organisational issues** |  |  |  |
| Emergency pass, information material |  |  |  |
| Membership DMG |  |  |  |
| Events/networking |  |  |  |
| Lifestyle and MG |  |  |  |
| Traveling and MG |  |  |  |
|  |  |  |  |
| **Social legislation inquiries** |  |  |  |
| Rehabilitation, degree of disability |  |  |  |
| Remedies and Aids |  |  |  |

5. Overview of topics of interest and inquiries to the DMG ‘before COVID-19 pandemic’

6: Overview of topics of interest and inquiries to the DMG ‘during COVID-19 pandemic’

7. Overview of topics of interest and inquiries to the iMZ ‘before COVID-19 pandemic’

8. Overview of topics of interest and inquiries to the iMZ ‘during COVID-19 pandemic’
